# Trying to Avoid Your Sister

**DOI:** 10.1371/journal.pbio.1000519

**Published:** 2010-10-19

**Authors:** Jessica P. Lao, Neil Hunter

**Affiliations:** Howard Hughes Medical Institute and the Departments of Microbiology, Molecular and Cellular Biology, and Cell Biology and Human Anatomy, University of California Davis, Davis, California, United States of America

## Abstract

During meiosis, the template for homologous recombination is not chosen at random as described in this Primer by Jessica Lao and Neil Hunter.

Connections between chromosomes are essential for their accurate segregation during cell division. In somatic cells dividing by mitosis, newly replicated sister chromatids are connected by cohesin proteins. When the sister chromatids become attached to microtubules emanating from opposite poles of the spindle, cohesins resist the pulling forces and the ensuing tension stabilizes the chromatid–microtubule attachments. In this way, each pair of sister chromatids achieves a stable bipolar attachment to the spindle. Consequently, when cohesion is destroyed at the onset of anaphase, sister chromatids are pulled to opposite poles and each new cell receives a full complement of maternal and paternal chromosomes.

In the germline, meiosis employs two successive rounds of nuclear division to produce gametes containing half the number of chromosomes as the original precursor cell. During the first division, sister chromatids remain connected while the paternal and maternal homologs are segregated (one homolog comprises a pair of sister chromatids). Homolog segregation during meiosis is governed by the same mechanical principles as sister segregation during mitosis and, as such, homologs must be connected. These connections are called chiasmata, and they are established via a process called homologous recombination, a DNA repair process that involves interaction between a broken chromosome and a homologous template chromosome.

To ensure that each pair of homologs is connected by at least one chiasma, homologous recombination during meiosis is regulated at several levels. A key aspect of this regulation is the choice of recombination template. The sister chromatid is the preferred template for recombinational repair in cells dividing by mitosis. However, during meiosis this bias must be overcome so that homologs recombine and become connected by chiasmata. How template choice is regulated remains unclear, but studies of meiotic recombination in budding yeast have suggested a number of possible mechanisms.

## Meiosis

**Figure 1:**
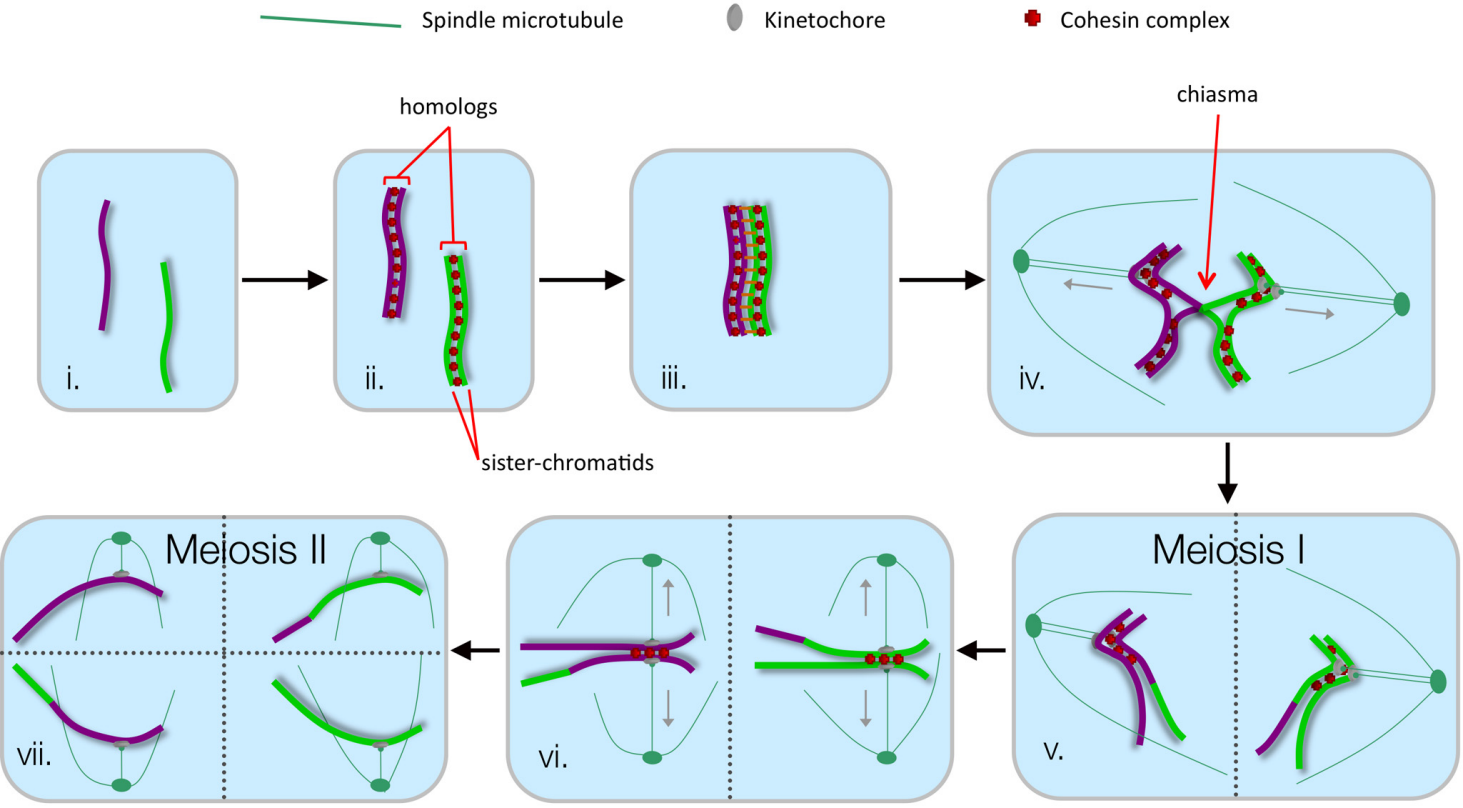
Meiosis. (i) Diploid cell with a single pair of homologous chromosomes (purple and green lines). Stages ii–iv; meiotic prophase. (ii) Chromosomes replicate to give pairs of sister chromatids connected by cohesion. (iii) Homologs pair and become synapsed along their lengths. Crossing-over occurs during this period. (iv) The resulting chiasma links the homologs and thereby facilitates stable bipolar attachment to the meiosis-I spindle. (v) Cohesion between the chromosome arms is lost and homologs are pulled to opposite poles. (vi) Maintenance of cohesion between centromeres allows bipolar attachment of sister chromatid pairs to the meiosis-II spindle. (vii) The remaining cohesion is lost and sister chromatids are segregated. Grey arrows indicate directions of the pulling forces generated by microtubules. Dashed lines indicate the planes of cell division.

In most organisms, meiosis produces haploid gametes from diploid precursor cells [1]. In this way, meiosis prevents the number of chromosome sets from doubling upon fertilization and thereby maintains the ploidy of a species with each successive generation. Meiosis halves the chromosome number via two successive rounds of chromosome segregation that follow a single round of chromosome replication (Figure 1). Homolog segregation during the first division is unique to meiosis and is achieved through two key processes: first, the parental homologs pair and become connected by one or more chiasma, the products of physical exchange (crossing-over) between two non-sister chromatids; second, the two kinetochores of each pair of sister chromatids behave as a single functional unit. Together with chiasmata, this “monopolar” behavior of sister kinetochores facilitates the bipolar attachment of homologs to the spindle such that homologs (not sister chromatids) are separated at the first meiotic division.

## The Roles of Homologous Recombination

Interactions between maternal and paternal homologs are the central theme of meiosis. While cohesin maintains the connections between newly replicated sister chromatids [2], connections between homologs must be established de novo. To this end, meiotic cells employ the chromosome repair process called homologous recombination [1]. The central reaction of recombination involves the pairing and strand exchange between a DNA strand, from an end of a broken chromosome, and a homologous template duplex. The resulting joint molecule (JM) intermediate, called a displacement loop (D-loop), provides a primer-template substrate for the new DNA synthesis required to repair the damaged chromosome.

Meiotic cells induce recombination by forming numerous programmed DNA double-strand breaks (DSBs). In most organisms the homology-dependent DNA-pairing aspect of recombination then mediates the two-by-two association of the parental homologs that culminates in the intimate synapsis of the homolog pairs along their entire lengths (Figure 1iii). Subsequently, crossovers, in combination with sister-chromatid cohesion, form the chiasmata required for accurate homolog disjunction at the first division (Figure 1iv).

## The Problem of Template Choice

**Figure 2:**
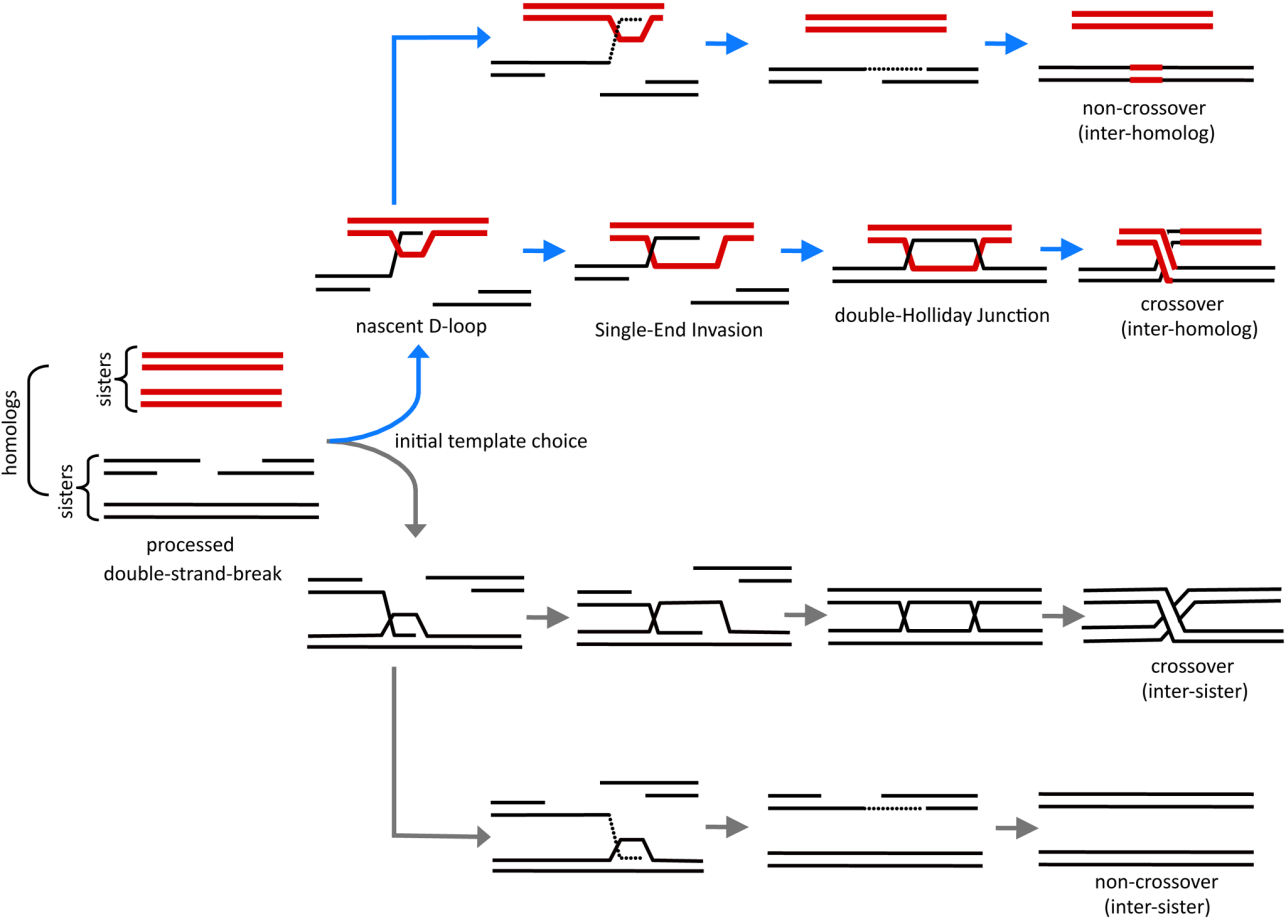
Pathways of meiotic recombination. The size difference between duplexes from the two homologs represents restriction-site polymorphisms that have been engineered at specific loci and utilized to monitor meiotic recombination intermediates by molecular assays [11],[12],[15] (see Figure 3). Dashed lines indicate new DNA synthesis. SEIs comprise a DSB end and a homologous duplex [12], but their exact structure remains uncertain. dHJs can also be resolved to produce non-crossover products, but resolution into crossovers appears to predominate during meiosis.

Meiotic recombination occurs during a protracted G2 period that follows DNA replication (stages ii to iv in Figure 1). Thus, no fewer than three allelic templates are available for recombination—the two homologs and one sister chromatid (see Figure 1ii and Figure 2). In order for recombination to be productive for pairing and chiasmata formation it must occur between homologs. Given the 2∶1 odds in favor of homolog templates, this might seem to be an insignificant problem. However, in cells dividing by mitosis, recombinational repair of DSBs preferentially utilizes the sister template [3]–[5]. This “against the odds” template bias is important for genome stability because allelic inter-sister recombination prevents the potentially deleterious effects of recombination such as loss of heterozygosity, chromosome rearrangements, and missegregation. The intrinsic inter-sister bias of recombination in mitotic cells appears to be promoted by the cohesin-dependent proximity of sister chromatids [6]. Thus, during meiosis, sister-chromatid cohesion can be viewed as a double-edged sword: it is essential for the formation of functional chiasmata at the end of prophase, but by favoring the sister template it opposes inter-homolog recombination during early prophase. In actuality, meiotic recombination is clearly biased towards homolog templates in most organisms (but see [7]). In budding yeast, estimates of inter-homolog bias range from 3∶1 to more than 7∶1, and this bias is reversed in a number of mutant situations indicating that it is the consequence of an active process that somehow resists an intrinsic tendency for inter-sister recombination [8]–[13].

## The Molecular Mechanism of Meiotic Recombination

The ends of meiotic DSBs are rapidly processed to form long single-stranded tails that serve as substrates for assembling filaments of the RecA-family proteins, Rad51, which is ubiquitously expressed, and Dmc1, which is meiosis-specific (Figure 2) [14]. The resulting nucleoprotein filaments mediate the search for homology and catalyze DNA strand exchange to form JM intermediates. Meiotic DSBs are ultimately repaired with one of two outcomes: a crossover with exchange of chromosome arms (leading to chiasma formation), or a non-crossover. Crossover and non-crossover pathways are distinct and appear to differentiate shortly after the initial strand exchange [12],[15],[16]. Along the crossover pathway, two major types of JM have been identified in vivo (Figure 2): single-end invasions (SEIs), in which one DSB end has undergone strand exchange with a template chromosome; and double Holliday Junctions (dHJs) in which both DSB-ends have been engaged [12],[17]. Non-crossovers are thought to arise primarily via the synthesis-dependent strand-annealing pathway, in which the invading DSB end is extended by DNA synthesis and then dissociated from the template, before being annealed to the other DSB end [18],[19]. The predicted D-loop non-crossover intermediates have not been identified in vivo, probably because they are less stable and shorter lived than SEIs and dHJs. Similarly, along the crossover pathway, SEIs appear relatively late in prophase, after homologs have paired (stage iii in Figure 1) [12] (N.H., unpublished observations). This implies that pairing is preceded and mediated by nascent D-loops that remain, as yet, undetected.

## Monitoring Template Choice

**Figure 3:**
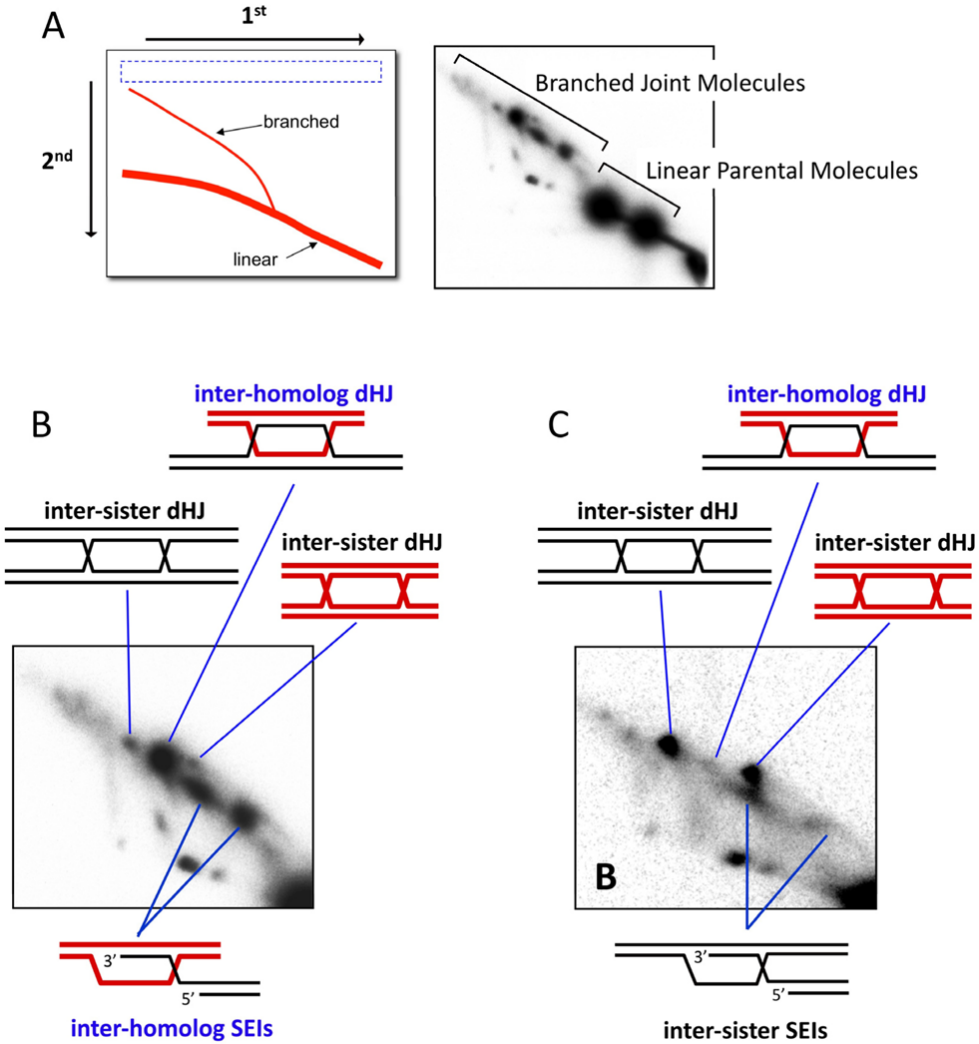
Monitoring template choice by 2-D gel electrophoresis. (A) The second dimension of a 2-D gel accentuates the shape element of DNA molecules such that branched species migrate more slowly than linear duplexes of identical mass. The right hand panel shows detection of JM intermediates via Southern hybridization of a 2-D gel. The analyzed locus contains restriction-site polymorphisms between the two parental homologs. (B) Close-up of the JMs in (A), highlighting the SEI and dHJ intermediates. Note the preponderance of inter-homolog dHJs relative to the inter-sister dHJs. (C) 2-D gel analysis of a mutant with a defect in template choice. In this strain, inter-homolog dHJs are almost absent and nearly all JMs, both SEIs and dHJs, are formed between sister chromatids.

Sister chromatids are identical and, as such, allelic recombination between sister chromatids is very hard to monitor. The only direct assay that has been routinely applied to measure template choice during meiosis is two-dimensional (2-D) gel electrophoresis of JM intermediates [20] (Figure 3; to date, this approach has only been applied to studies of recombination in yeast). When suitable restriction fragment length polymorphisms are engineered into the chromosomes, Holliday Junction containing JMs (dHJs and/or single-HJs) formed between homologs or sisters can be distinguished based on their relative molecular weight and migration behavior in the second dimension (branched DNA molecules migrate more slowly than linear molecules of the same mass) [10].

## Factors Implicated in Template Choice during Meiosis

Mutations in a number of genes diminish meiotic inter-homolog bias in budding yeast. Most, though not all, of these genes appear to be broadly conserved suggesting that the basic mechanisms underlying inter-homolog bias are also conserved. These genes fall into two distinct functional categories:

1. Components of a phosphokinase signal transduction pathway that responds to meiotic DSBs and modulates recombination and progression through meiotic prophase [21]. This pathway includes core DNA damage response factors, such as the sensor kinases, Mec1/Tel1, as well as meiosis-specific components Hop1, Red1, and Mek1 [1],[10],[11],[22]–[27]. Red1 and Hop1 assemble along meiotic chromosomes into ensembles that mediate signaling between DSB sensor kinases (Mec1/Tel1) and the meiosis-specific serine/threonine effector kinase, Mek1.
2. Factors involved directly in DNA strand exchange including Rad51, Dmc1, and several associated factors [11],[28],[29]. For example, when Rad51 is mutated, Dmc1-dependent recombination occurs primarily between sister chromatids.

Several studies have demonstrated genetic interactions between mutations in these two categories of genes. For example, *dmc1* mutants arrest in meiotic prophase with unrepaired DSBs, but additional mutation of Hop1, Red1, or Mek1 alleviates this arrest. In these cases DSBs are repaired, but repair occurs primarily via inter-sister recombination [1],[10],[11],[22],[23],[25]–[27],[30]. These phenotypes are explained by the fact that the Mek1-kinase inhibits Rad51-mediated strand exchange when Dmc1 is absent [31]. It is tempting to think that inhibition of Rad51 during meiosis helps to counteract the tendency of the core mitotic recombination machinery to utilize the sister template.

## How Could Interhomolog Bias Work?

If meiotic DSB repair were allowed to proceed unchecked, the expectation is that most DSBs will be rapidly and unproductively repaired using the sister template. This expectation is borne out by analysis of mutations in the Hop1–Red1–Mek1 pathway [11],[13],[25],[32]. Thus, inter-homolog bias must somehow be actively imposed.

The behavior of *dmc1* mutants (described above) has led to the idea that a barrier to inter-sister recombination is established during meiosis, essentially forcing the use of homolog rather than sister templates [22],[27],[32]. This idea is supported by the observation that DSB repair is very inefficient in haploid yeast cells, in which homologs are absent and inter-sister recombination is the only option (haploid yeast cells don't normally do meiosis, but they can be “tricked” into doing so) [27]. It is important to note that this barrier must be imposed locally, on a DSB-by-DSB basis because a general block to inter-sister recombination would also constitute a general block to inter-homolog recombination.

Numerous studies make it clear, however, that the sister template is available (or becomes available) for recombination during meiosis. In this issue of *PLoS Biology*, Goldfarb and Lichten provide direct evidence that the sister template is used efficiently for meiotic DSB repair when allelic homolog templates are absent. They also infer that inter-sister repair may occur much more frequently than previously estimated from analyses of JM intermediates. This conclusion echoes previous inferences that the sister template is frequently engaged during meiotic recombination [33],[34].

Thus, in wild-type cells, any barrier to inter-sister recombination appears to be, at most, transient. Counter to the idea of a barrier, Goldfarb and Lichten suggest a “kinetic impediment” model in which Mek1 promotes inter-homolog bias by specifically slowing down the normally faster rate of inter-sister recombination, such that the rates of inter-sister and inter-homolog recombination are now effectively equalized. This idea is consonant with the established observation that inter-sister and inter-homolog JMs form with identical timing [10],[11], and reconciles the accelerated rate of DSB-repair measured in *mek1* mutants. Under this model, the block to inter-sister repair observed in *dmc1* mutants and in haploid cells (described above) is proposed to reflect a general block to recombination caused by pathological pan-nuclear hyperactivation of Mek1.

The “barrier” and “kinetic impediment” models are broadly similar in their basic premise that by negatively regulating inter-sister recombination, inter-homolog recombination is promoted as the only possible alternative. Contrasting, albeit non-exclusive, models propose that inter-homolog recombination is positively regulated. Such models do not dictate that access to the sister chromatid be blocked per se, but that inter-homolog bias is implemented by preferentially promoting inter-homolog interactions [1],[11],[28],[30]. This could be achieved, for example, by making the stabilization of nascent JMs (and/or their progression to later steps) dependent upon the development of inter-homolog interactions (i.e., pairing and synapsis).

## Why the Sister Template Is Important for Meiotic Recombination

Unrepaired DSBs are fatal. Therefore, in addition to the primary goals of homolog pairing and chiasmata formation, the meiotic cell must ensure that all DSBs are efficiently repaired. The logical way to accomplish this is to use all available templates, the homologs and the sister. Goldfarb and Lichten [13] highlight the importance of the sister template when parental chromosomes are heterozygous for commonly occurring chromosomal rearrangements such as insertions/deletions (but also translocations or inversions, or even when allelic homology is low, termed homeology). In these situations, inter-homolog strand exchange will not be possible, and repair via the sister template becomes essential for viability. In fact, the standard karyotypes of most organisms dictate that sister chromatid recombination is essential during meiosis. For example, although recombination between mammalian X and Y chromosomes can only take place between small stretches of shared homology, called the pseudoautosomal regions, DSBs form along the length of the X chromosome [35]. Similarly, an absolute requirement for the sister template must occur in males of species with the Protenor mode of sex determination (X = male; XX = female or hermaphrodite).

More generally, the sister chromatid may regularly be engaged to more efficiently complete recombination [34]. For example, inter-homolog strand exchange events that initially function to facilitate homolog pairing could subsequently be dissociated, and repair completed via recombination with the sister chromatid.

## Goals for the Future

To ensure that each pair of homologs becomes connected by chiasmata, meiotic recombination must be regulated at multiple levels: (i) DSB formation, to ensure that recombination is initiated on all homologs; (ii) template choice, to favor inter-homolog interactions; (iii) the crossover/non-crossover outcome, to produce at least one crossover; and (iv) spatial–temporal integration with the other events of meiotic prophase, i.e., homolog pairing, synapsis and segregation. Despite stunning progress in recent years, our understanding of these regulatory processes remains vague.

Currently, the analysis of template choice during meiotic recombination in yeast is limited by the fact that only relatively late-arising, metastable JMs can be monitored. The levels and ratios of these JMs do not necessarily provide an accurate readout of the initial template choice made during the critical period when homologs are being paired. Moreover, several lines of evidence indicate that a single DSB end can engage different templates, perhaps multiple times, before forming a stable JM or recombinant product, e.g., [34],[36]. Thus, it remains possible that recombination is strongly biased towards homolog templates during early stages of meiotic prophase. Therefore, in order to fully understand the complexities of template choice during meiotic recombination, methods to monitor *initial* recombinational interactions must be developed.

Understanding the regulation of template choice in organisms other than yeast remains a major challenge. Cytological approaches that allow the visualization of inter-homolog and inter-sister crossovers [37] could be used to analyze mutants inferred to be defective for template choice, but ultimately the development of techniques to detect all products of recombinational repair (inter-homolog, inter-sister, crossover and non-crossover) will be required.

## Abbreviations

2-D: two-dimensional
DSB: double-strand break
D-loop: displacement loop
JM: joint molecule
SEI: single-end invasion
dHJ: double Holliday Junction
